# State Help for Investigations into Contagious Diseases of Animals

**Published:** 1881-01

**Authors:** 


STATE HELP FOR INVESTIGATIONS INTO THE CONTAGIOUS DISEASES OF ANIMALS.—Very striking inferences regarding the nature of *contagia vera* may be drawn from the experiments of Pasteur, Toussaint, and Greenfield, that have been just described. That is to say, we might draw such inferences if the experiments referred to were at all corroborated by others. There is great need that such confirmation be furnished, if possible. And if it is proved that the "vaccine" of the two contagious diseases already investigated can be obtained by artificial processes, there should be further experiments to extend the application to other diseases. It is very desirable that these and kindred questions in comparative pathology be taken up on this side of the Atlantic. We lack greatly the official aid which men and institutions abroad receive so generally. There could be no greater help given to science and the interests of live stock owners than by direct appropriations for scientific researches, or, better, by establishing some institution like the Brown Institute in London, where work in comparative pathology could be carried on systematically. It is unfortunate that comparative pathology, though capable of throwing so much light upon disease, offers no pecuniary reward to those who would like to study it. All the more it deserves the help of an enlightened State.

On the other hand, the vast and constantly increasing interests of live stock owners demand the wisest care from our legislators. The loss of property occasioned by diseases, contagious and otherwise, among animals, is immense, and deserves much more attention than it has ever yet received. If there are any methods by which these diseases can be prevented, cured, or stamped out, they should be known. Such methods can only be learned, however, by patient study and investigation—work which cannot be prosecuted without State help. This it should receive.

